# Polypyrrole Polyethylene Composite for Controllable Linear Actuators in Different Organic Electrolytes

**DOI:** 10.3390/ma15020540

**Published:** 2022-01-12

**Authors:** Nguyen Quang Khuyen, Ngoc Tuan Nguyen, Rudolf Kiefer

**Affiliations:** 1 Conducting Polymers in Composites and Applications Research Group, Faculty of Applied Sciences, Ton Duc Thang University, Ho Chi Minh City 700000, Vietnam; nguyenquangkhuyen@tdtu.edu.vn; 2 Faculty of Applied Sciences, Ton Duc Thang University, Ho Chi Minh City 700000, Vietnam; nguyenngoctuan@tdtu.edu.vn

**Keywords:** PPy-PEO, PPy/DBS, linear actuation, three different electrolytes, controllable actuation

## Abstract

Controllable linear actuation of polypyrrole (PPy) is the envisaged goal where only one ion dominates direction (here anions) in reversible redox cycles. PPy with polyethylene oxide (PEO) doped with dodecylbenzenesulfonate forms PPy-PEO/DBS films (PPy-PEO), which are applied in propylene carbonate (PC) solvent with electrolytes such as 1-ethyl-2,3-dimethylimidazolium trifluoromethanesulfonate (EDMICF_3_SO_3_), sodium perchlorate (NaClO_4_) and tetrabutylammonium hexafluorophosphate (TBAPF_6_) and compared in their linear actuation properties with pristine PPy/DBS samples. PPy-PEO showed for all applied electrolytes that only expansion at oxidation appeared in cyclic voltammetric studies, while pristine PPy/DBS had mixed-ion actuation in all electrolytes. The electrolyte TBAPF_6_-PC revealed for PPy-PEO best results with 18% strain (PPy/DBS had 8.5% strain), 2 times better strain rates, 1.8 times higher electronic conductivity, 1.4 times higher charge densities and 1.5 times higher diffusion coefficients in comparison to PPy/DBS. Long-term measurements up to 1000 cycles at 0.1 Hz revealed strain over 4% for PPy-PEO linear actuators, showing that combination of PPy/DBS with PEO gives excellent material for artificial muscle-like applications envisaged for smart textiles and soft robotics. FTIR and Raman spectroscopy confirmed PEO content in PPy. Electrochemical impedance spectroscopy (EIS) of PPy samples revealed 1.3 times higher ion conductivity of PPy-PEO films in PC solvent. Scanning electron microscopy (SEM) was used to investigate morphologies of PPy samples, and EDX spectroscopy was conducted to determine ion contents of oxidized/reduced films.

## 1. Introduction

Conducting polymer (CP) actuators have been in research for nearly over 30 years with the aim at first to understand how actuation can be controlled and applied. Several directions of applications have been made such as microactuators [1] including microrobotic devices [2], biomedical devices [3] and recently smart textiles [4], as well many more [5]. The simplified mechanism of conducting polymer-based reversible volume changes (actuation) on the charging/discharging process leads to a delocalized positive charge in the CP film with counterions (anions) and solvent molecules (included osmotic balance [6]) entering at oxidation, called anion-driven actuation that led to a swelling of the CP and at reversed scan to shrinking at reduction (egress of anions and solvent if the CP is reduced). The other case of cation-driven actuation is immobile anions such as DBS^−^ incorporated during electropolymerization forming PPy/DBS films leading to expansion at reduction, also named cation-driven actuation. Those specified types do not comply if different electrolytes [7] or solvents [8] are applied, leading in most cases to the unfavorable mixed-ion actuation or changes in actuation directions (in the special case of PPy/DBS becoming anion-driven in propylene carbonate electrolyte [9]). Several effects in the electropolymerization process [10] such as temperature [11] and solvents [12], different electrolytes in the actuation process [13] and different applied potential windows [14] result in changes of actuation, making real application of conducting polymers difficult to control. 

Mixed-ion actuation has been shown from past research regarding PPy either made in aqueous [8] or in organic solvents [15]; it reduces the overall strain as well leads to the uncontrollable performance of those actuators. Mixed actuation is assumed to take place because some applied electrolytes, mainly anions, stay immobile in the PPy network, leading to partial expansion at reduction [15]. The reason why such anions stay inside the PPy network is the changing of sigma-bonds (cross-linkage) during reversible redox cycles keeping former mobile anions immobile [16] or in other cases the anions having a nonspherical form such as triflate anions (CF_3_SO_3_^−^) not being able to diffuse out at reduction, shown especially on PEDOT linear films [17] where the porosity is greater than in PPy films. The ion mobility inside PPy films can be enhanced by increasing the overall conductivity of the films, for example by adding additional conductive particles as in ion gold implementation [18] or including polyelectrolytes to enhance ion conductivities [19,20]. PEO is known to be a polyelectrolyte often applied in Nafion membranes to increase ion mobility for lithium battery applications [21] but is also applied in bending actuators that contain an IPN membrane with PEO, sandwiched between conducting polymer electrodes [22].

Previous research using PEO in electropolymerization did reveal that PPy-PEO formed films lead to enhanced ion conductivity, and the 5 wt.% load was found optimal [23]. Nevertheless, the effects of different electrolytes in propylene carbonate solvent have not been shown for PPy-PEO films. Electrolytes such as EDMICF_3_SO_3_, NaClO_4_ and TBAPF_6_ which have the tendencies of mixed-ion actuation shown in PPy films [24,25] are designated. The linear actuation response of PPy-PEO is compared to pristine PPy/DBS films. In one case we want to demonstrate that mixed-ion actuation can be suppressed by adding PEO, and in another case we want to evaluate which electrolyte gives the best response in achieving a high strain adaptable and controllable in actuation direction for future envisaged applications of artificial muscle-like actuators.

Cyclic voltammetry and square potential steps combined with linear actuation measurements were performed with determination of diffusion coefficients to evaluate which anions move faster with included long-term measurements of the favorite electrolyte. SEM images of surface and cross-section of samples were made and the electronic conductivity was compared between nonactuated and actuated samples. FTIR spectroscopy of PPy/DBS and PPy-PEO was compared and Raman spectroscopy was conducted to determine the doping state of PPy samples directly after polymerization. EIS measurements were performed on PPy samples in PC solvent to determine the ion conductivity. EDX spectroscopy of PPy-PEO and PPy/DBS films after actuation were analyzed to evaluate which elements can be found in oxidized and reduced samples.

## 2. Experimental

### 2.1. Chemicals

1-Ethyl-2,3-dimethylimidazolium trifluoromethanesulfonate (EDMICF_3_SO_3_, 95%), tetrabutylammonium hexafluorophosphate (TBAPF_6_, >99%), sodium persulfate (NaClO_4_, >98%), sodium dodecylbenzenesulfonate (NaDBS, 99%), polyethylene oxide (PEO, M_v_ 100.000 g mol^−1^), ethylene glycol (EG, 99.8%), propylene carbonate (PC, 99%) and ethanol (technical) were purchased from Sigma-Aldrich (Taufkirchen, Germany) and used as supplied. Pyrrole obtained from Sigma Aldrich was distilled at reduced pressure and stored at low temperature (−20 °C) under nitrogen. Deionized water Milli-Q+ (Tallinn, Estonia) was used as supplied.

### 2.2. Electropolymerization

The monomer solution included 0.1 M NaDBS and 0.1 M pyrrole in aqueous EG: Milli-Q+ (50:50 wt.%) and formed PPy doped with DBS^−^ films in galvanostatic electropolymerization (0.1 mA cm^−2^, 11.1 h time) at the low temperature of −20 °C. The polymerization took place in a three-electrode setup using a stainless steel sheet (12 cm^2^) where the PPy was deposited on each side opposite a stainless steel mesh (counter electrode) and an Ag/AgCl wire as a reference electrode. The formation of PPy-PEO films was conducted in a similar process using pristine PPy/DBS with the addition of 5 wt.% PEO in a monomer solution. Both PPy samples were washed several times with ethanol to remove excess pyrrole and were washed with Milli-Q+ to remove excess of the electrolyte NaDBS and PEO on the surface of samples. The PPy films were then dried in the oven at 40 °C (2 mbar) for 24 h and applied for further characterizations. The PPy/DBS and PPy-PEO samples were stored in different electrolytes applied in this work, and the thickness was determined with an electronic Gauge Meter (Coolant Proof Micrometer Series 293, 0.001 mm sensitivity, Mitutoyo America Corporation, Aurora, IL, USA). The average thickness of all samples was in the range of 30 ± 2 µm.

The PPy/DBS and PPy-PEO films were cut in 1 cm length and 0.1 cm width. The PPy films are fixed between the upper clamp connecting to the force sensor (TRI202PAD, Panlab, Barcelona, Spain) and the lower clamp of a fixed arm containing gold contacts (working electrode) in the three electrodes electrochemical cell with platinum sheet counter electrode and a Ag/AgCl (3 M KCl) reference electrode in the home-made linear muscle analyzer set-up [26]. The experimental set-up is presented in Scheme S1. The potentiostat (Biologic PG581, Seyssinet-Pariset, France) was connected with the linear muscle analyzer giving the changes in mass or length in real time accompanied with in-house software [26]. The PPy films were stretched up to 1% (constant force of 29.4 mN) 12 h before measurements commenced. PPy/DBS and PPy-PEO film length changes were conducted in 0.2 M concentration of three different electrolytes using PC as solvent (EDMICF_3_SO_3_, NaClO_4_ and TBAPF_6_) applying cyclic voltammetry and square potential steps at frequency range 0.0025 Hz to 0.1 Hz. The diffusion coefficients were calculated (Equations (1) and (2) [27]) from current density time curves at each applied frequency at oxidation under the condition of the electrochemically stimulated conformational relaxation (ESCR) model [28].

(1) $$\ln\left[1-\frac{Q}{Q_{t}}\right]=-b\cdot t$$

(2) $$D=\frac{b\cdot h^{2}}{2}$$

The left side of Equation (1) contains the charge at each time obtained over the integration of current density time curves divided by the total charge *Q_t_* and given in the expression plotting against time *t* gives a curve where the slope *b* was determined [29]. With the thickness *h* of the film samples, the diffusion coefficient (Equation (2)) was calculated. At least three samples of each PPy-PEO and PPy/DBS film were polymerized and independently measured in different electrolytes. The results are shown as mean values with standard deviations.

### 2.3. Characterizations

SEM images of surface and cross-section at 10 kV (Helios NanoLab 600, FEI, Hillsboro, OR, USA) of PPy/DBS and PPy-PEO films in dry state directly after polymerization were made. FTIR (4000–600 cm^−1^. Bruker Alpha with Platinum ATR, Billerica, MA, USA) and Raman spectroscopy (Renishaw System 1000 microprobe, 785 nm excitation line, Wotton-Under-Edge, UK) of PPy-PEO and PPy/DBS films were conducted. The EIS measurements (alternative current between 10 mHz and 2 MHz, PARSTAT 2273 potentiostat FRA, Princeton Applied Research, Berwyn, PA, USA) of PPy samples (length of 1.0 cm and width of 0.5 cm, area *A*: 5 × 10^−5^ m^2^, thickness *w*: 30 μm) were performed in PC solvent to obtain *Z_re_* (real part of polymer PPy impedance) from the Nyquist plot to calculate the ion conductivity *σ_i_* regarding Equation (3).

(3) $$\sigma_{i}=\frac{w}{Z_{re}\cdot A}$$

The ion content of the cross-section samples was determined by EDX spectroscopy (EDX with X-Max 50 mm^2^ detector, Oxford Instruments, High Wycombe, UK) after actuation cycles of PPy films at oxidized state (1 min, 1.0 V) and reduced state (1 min, −0.55 V). The surface conductivity (at oxidation in dry state) before and after actuation cycles in different electrolytes was determined using four-point-probe conductivity meters (Jandle 4-Point Probe Head, Model RM2, Leighton Buzzard, UK).

## 3. Results and Discussion

The general trend in current research is to focus on applications, while mechanistic studies are not followed up. To control actuators, the mixed-ion actuation appeared in several studies to lower the overall strain outcome [15], or if in the same size at oxidation/reduction, special design can present new application [30]. Previous research [31] revealed that the typical cation-driven actuators PPy/DBS changed to mainly anion-driven in organic electrolytes. Addition of PEO in electropolymerization led to enhanced ion conductivity [23,32]. We want to demonstrate that the mixed-ion activity can be reduced in PPy-PEO linear actuators in comparison to PPy/DBS types, leading to high strain adaptability as an “artificial muscle” candidate.

### 3.1. Characterization of PPy/DBS and PPy-PEO Films

The PPy/DBS and PPy-PEO films were characterized by SEM in surface and cross-section images to evaluate if there are any differences in morphology. FTIR and Raman spectroscopy of samples were conducted to determine the incorporation of PEO and compare the doping state of both PPy films. EIS measurements of samples in the applied different electrolytes were performed to reveal the ionic conductivity, and EDX spectroscopy gave the ion content at oxidation/reduction of PPy/DBS and PPy-PEO after actuation cycles.

#### 3.1.1. Electropolymerization, SEM Images and Electronic Conductivity

**Figure 1:**
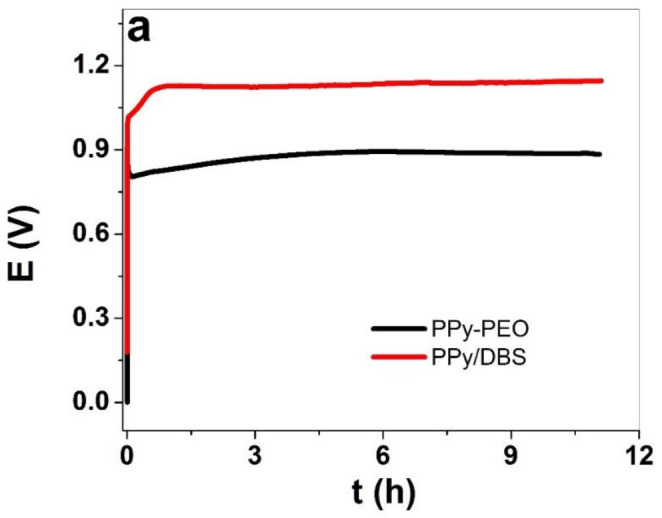

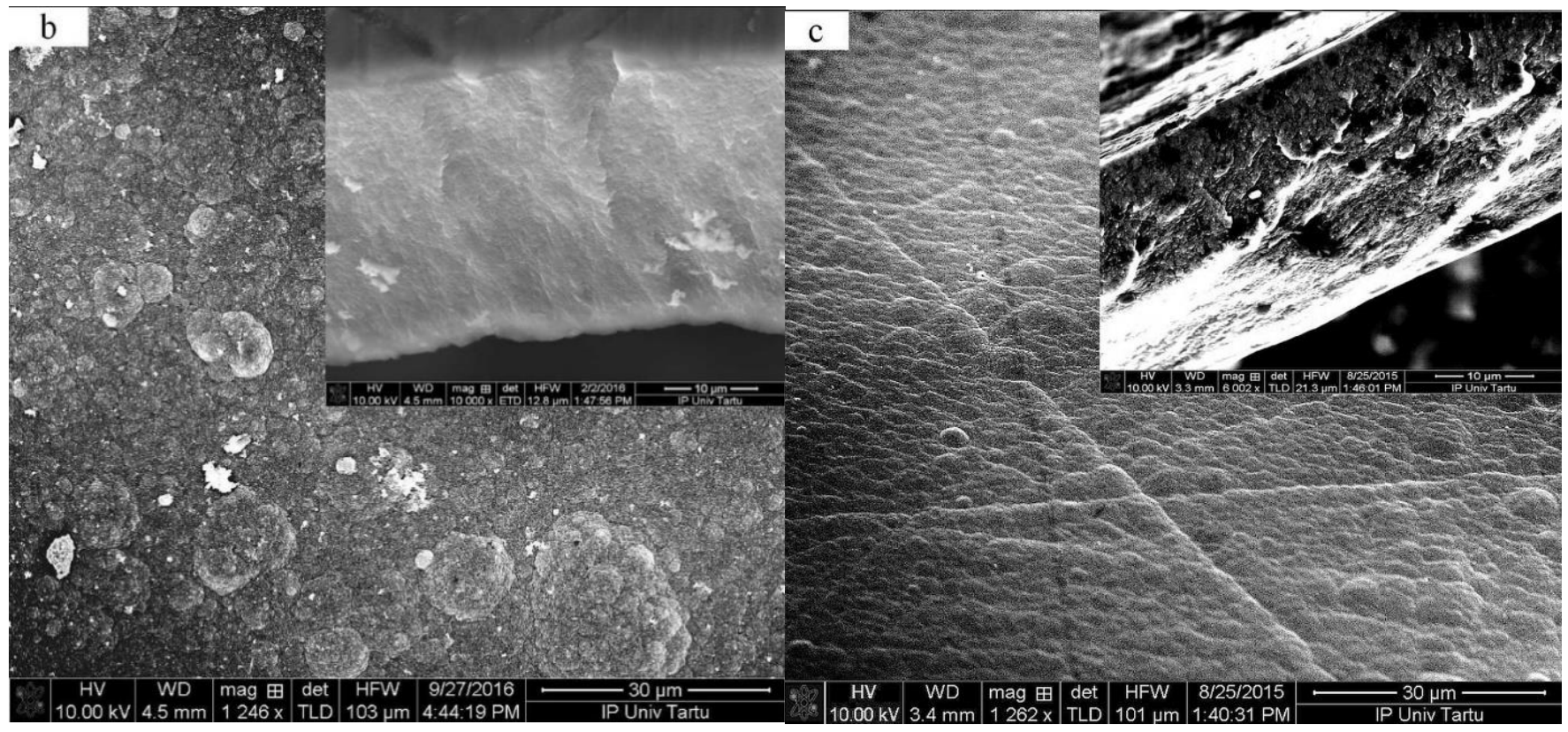
Galvanostatic electropolymerization (0.1 mA cm^−2^, −20 °C, 11.1 h) of PPy/DBS (red curve) and PPy-PEO (5 wt.% PEO, black curve) showing (**a**) potential E against time t. The SEM surface images (scale bar 30 μm) with inset of cross-section image (scale bar 10 μm) showing (**b**) PPy-PEO and (**c**) PPy/DBS films.

The electropolymerization curves of PPy/DBS and PPy-PEO are shown in Figure 1a, and the SEM images of the surface with inset cross-section are presented in Figure 1b,c.

The PPy/DBS galvanostatic electropolymerization (Figure 1a) shown on the end of the polymerization curve a potential of 1.1 V, and PPy-PEO had 0.89 V (nearly 19% reduction). The electropolymerization was studied extensively in the past [33] with the main principle that the monomer pyrrole standard potential is the first initial step to oxidize forming radical cations which combine with other cations to form dimers until a certain chain length is achieved (oligomers), and the oligomers then became insoluble in the electrolyte and deposit on the stainless steel working electrode. The slower the process, which can be achieved at reduced temperature [11], the more compact and dense the PPy films appear. The addition of PEO, i.e., ion-conductive polymer chains, in electropolymerization has an effect in increasing ion conductivity of the monomer solution [34], which will affect the ion conductivity of the deposited PPy-PEO film, lowering the potential in electropolymerization. The SEM surface images of PPy-PEO (Figure 1b) reveal the typical cauliflower structure [35] with much rougher morphology in comparison to PPy/DBS (Figure 1c), and it was shown from former research that increasing PEO content led to more swollen morphology [23]. The cross-section image of PPy-PEO (inset Figure 1b) in comparison to PPy/DBS (inset of Figure 1c) appeared to be more dense and compact. The electronic surface conductivity of PPy/DBS and PPy-PEO films directly after polymerization and after actuation cycles in the three different electrolytes is presented in Table 1.

The electronic conductivities of pristine PPy-PEO were 1.8 times higher for PPy/DBS type (Table 1). After actuation studies in different electrolytes, the electronic conductivities increased, as also shown from previous studies for PPy/DBS films [7]. In the case of PPy-PEO films, the same tendency was observed, with the best electronic conductivity found for TBAPF_6_-PC followed by EDMICF_3_SO_3_-PC electrolytes. For PPy/DBS and PPy-PEO, the lowest conductivity was found in the NaClO_4_-PC electrolyte (Table 1).

#### 3.1.2. FTIR, Raman and EIS Spectroscopy

**Figure 2:**
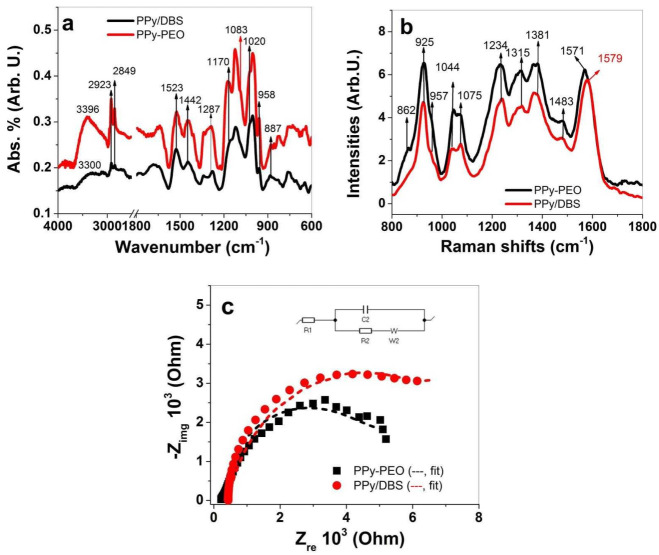
FTIR spectroscopy (4000−600 cm^−1^) of PPy-PEO (black line) and PPy/DBS (red line) is shown in (**a**) and Raman spectroscopy (785 nm, 1800–800 cm^−1^) is presented in (**b**). The EIS measurements of PPy samples conducted in PC solvent of PPy-PEO (■, -- fit) and PPy/DBS (●, --) are shown in (**c**). The inset in (**c**) represents Randle’s equivalent circuit where R1 is the solution resistance, R2 represents the charge transfer resistance, C2 describes the double-layer capacitance and W2 is the Warburg element.

To investigate the composition of PPy/DBS and PPy-PEO directly after polymerization (in oxidized state at 1.0 V), FTIR and Raman spectroscopy were performed, with results shown in Figure 2a,b, respectively. The results of EIS measurements in PC solvent are presented in Figure 2c.

FTIR signals of PPy/DBS and PPy-PEO (PPy-PEO/DBS) shown in Figure 1a revealed typical PPy/DBS signals [36,37,38] with broad waves at 3400−3300 cm^−1^ representing the N-H stretching. The double peaks at 2923 cm^−1^ and 2849 cm^−1^ refer to –CH_3_ and –CH_2_ stretching vibration of the immobile DBS^−^ anions in PPy. The 1523 cm^−1^ peak belongs to C=C stretching, and the 1442 cm^−1^ peak represents the C–N stretching vibration of the PPy ring. The C–N in-plane deformation of PPy is shown at 1287 cm^−1^, and the doping state of PPy [37] (C–H out of plane vibrations) is shown at 958 cm^−1^ and 887 cm^−1^. The peaks at 1170 cm^−1^ and 1020 cm^−1^ represent the symmetric and asymmetric stretching bands of S=O (DBS^−^ anions) [39]. The shoulder at 1083 cm^−1^ only shown in PPy-PEO belongs to the C–O–C stretching band of PEO [40].

Raman peaks of PPy [41,42,43] (Figure 2b) can be found at 1571 cm^−1^ (PPy-PEO) with a shift to 1579 cm^−1^ for PPy/DBS that represents the C=C backbone stretching. The shift in such peaks represents the conductivity of the samples, and shifts to lower frequency shown from previous research [41] refer to a higher oxidation state and therefore an increase in conductivity (Table 1) of PPy-PEO. Additional peaks representing PPy can be found at 1483 cm^−1^ (skeletal band), 1381 cm^−1^ and 1315 cm^−1^ (C–N stretching mode of PPy), and the 1234 cm^−1^ peak was identified as C–H in-plane bending. The double peaks of C-H in-plane bending at 1044 cm^−1^ and 1075 cm^−1^ represent the polaron/bipolaron content, and their ratios give evidence of the doping state of the samples [42]. The ratio for PPy/DBS found at 0.93 and those of PPy-PEO in the range of 1.1 confirmed that PPy-PEO directly after polymerization was more doped than PPy/DBS. The shoulder at 957 cm^−1^ (radical cation, polaron) and the peak at 925 cm^−1^ (dication, bipolaron) are another pair where the doping level (ring deformations) can be determined by their ratios, found for PPy/DBS at 0.5 and PPy-PEO at 0.61, showing the same tendency of PPy-PEO having a higher doping level. The 862 cm^−1^ shoulder (symmetric rocking mode of ethylene [44]) was only found in PPy-PEO samples and revealed the inclusion of PEO in PPy.

The EIS measurements in PC solvent of PPy films directly after polymerization (Figure 2c) revealed Z_re_ values of 5405 ± 212 Ω for PPy/DBS, and by putting those values in Equation (3) the ion conductivity was calculated as 1.1 ± 0.04 μS/cm. In the case of PPy-PEO, the Z_re_ value was 4159 ± 185 Ω, which led to ion conductivity of 1.44 ± 0.06 μS/cm which was nearly 1.3 times higher than those in PPy/DBS. In summary, FTIR spectroscopy showed that all PPy signals as well those from DBS^−^ are shown in PPy/DBS and PPy-PEO, and PEO inclusion in PPy was also identified. Raman spectroscopy confirmed that PPy-PEO has a higher doping state than PPy/DBS, and EIS measurement revealed an enhancement of ion conductivity, shown as well from past research [32].

#### 3.1.3. EDX Spectroscopy

**Figure 3:**
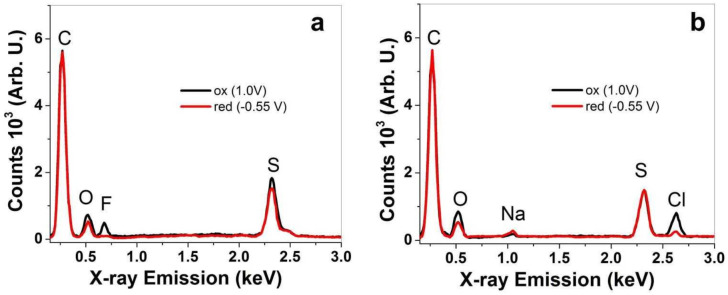

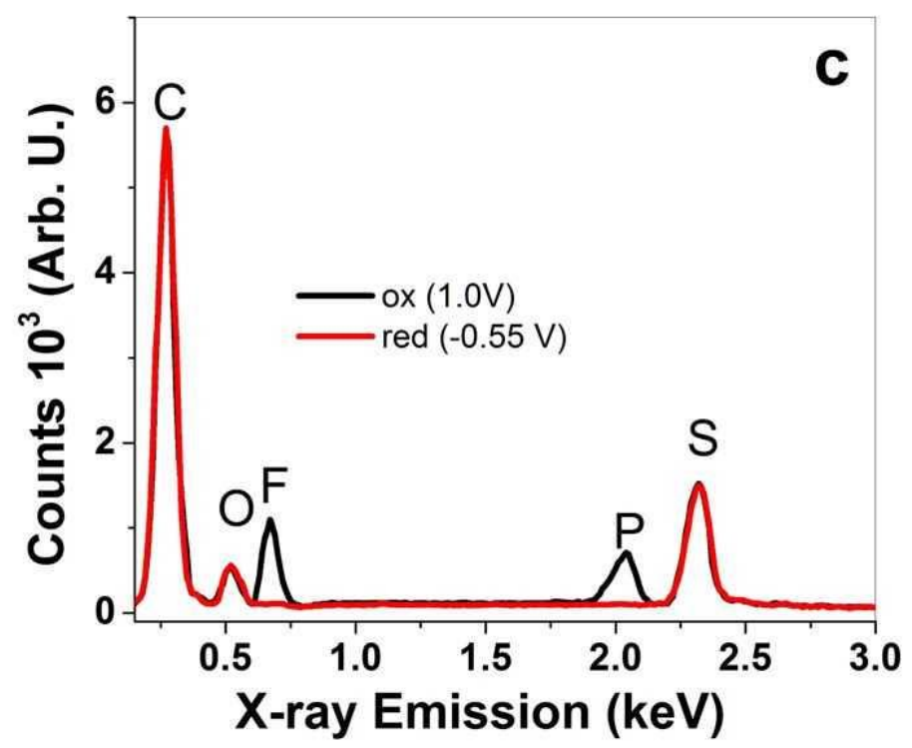
EDX spectroscopy (from cross-section images) of PPy-PEO films after actuation cycles at oxidized state (ox, 1 min, 1.0 V, black line) and reduced state (red, 1 min, −0.55 V, red curve) in different electrolytes: (**a**) EDMICF_3_SO_3_-PC, (**b**) NaClO_4_-PC and (**c**) TBAPF_6_-PC.

Additional investigation of PPy/DBS and PPy-PEO films regarding their ion content after actuation cycles in different electrolytes was performed using EDX spectroscopy. The results of PPy/DBS are shown in Figure S1a–c, and those from PPy-PEO are presented in Figure 3a–c.

In general EDX spectroscopy, here all spectra normalized on the carbon peak “C” at 0.27 keV show the basic components of PPy doped with DBS^−^ with oxygen “O” peak at 0.52 keV and sulfur peak “S” at 2.32 keV for PPy-PEO in Figure 3a–c and for PPy/DBS in Figure S1a–c. A slightly higher oxygen peak refers to PEO incorporation in PPy-PEO films [45]. The element composition after actuation at oxidation/reduction revealed a qualitative expression in which elements are detected in the PPy films. In the case of EDMICF_3_SO_3_-PC electrolyte, the fluoride peak “F” at 0.67 keV represents the anion CF_3_SO_3_^−^ shown in Figure 3a only at oxidation for PPy-PEO films (oxygen and sulfur peak are also increased). The PPy/DBS film in EDMICF_3_SO_3_-PC electrolyte (Figure S1a) revealed a small peak of fluoride at reduction, from which we assume that CF_3_SO_3_^−^ anions stayed immobile in the PPy/DBS film, resulting in a certain amount of expansion at reduction. With the stronger fluoride peak at oxidation, the anions are incorporated during oxidation. The EDX spectra (Figure S1a) give evidence for mixed-ion influence. The electrolyte NaClO_4_-PC in Figure 3b (shown for PPy/DBS in Figure S1b) had two additional elements, namely sodium “Na” at 1.04 keV and chloride “Cl” at 2.63 keV. For PPy-PEO (Figure 3b), there is a dominant chloride peak at oxidation with a stronger oxygen peak referring to ClO_4_ anions, while at reduction a small chloride peak remains with a minor sodium peak. In the case of PPy/DBS (Figure S1b), the sodium peak at reduction is much stronger, and sodium and chloride peaks are also found at oxidation. Therefore, the NaClO_4_-PC electrolyte has anion and cation movement in PPy films and is more pronounced for Ppy/DBS than Ppy-PEO. As shown in Figure 3c (Figure S1c, Ppy/DBS), the electrolyte TBAPF_6_-PC is applied in PPy-PEO films having a fluoride peak at 0.67 keV and a phosphor peak “P” at 2.04 keV, describing the anion PF_6_^−^. In the case of PPy-PEO films (Figure 3c), there is only PF_6_^−^ movement at oxidation, while in PPy/DBS (Figure S1c) small fluoride and phosphor peaks are retained at reduction [7]. There are molecules such as DBS^−^ and PEO which do not change during the oxidation/reduction process in the PPy samples. The different anions from the applied electrolytes give evidence of fluoride peaks, chloride peaks and phosphor peaks growing or shrinking during oxidation/reduction process to evaluate which ions are moving. Further analysis in linear actuation studies will reveal how the ion movements influence the linear actuation properties.

### 3.2. Linear Actuation Properties of PPy-PEO and PPy/DBS

Previous research [7] on PPy/DBS linear actuators revealed that in most applied electrolytes small mixed-ion effects took place while major expansion at oxidation led to main actuation properties. Even minor expansion at reduction led to reduced performance of the linear actuators. Cyclic voltammetry and square potential step measurements were performed. At least three samples of each PPy-PEO and PPy/DBS film were polymerized and independently measured in different electrolytes. The results are shown as mean values with standard deviations.

#### 3.2.1. Cyclic Voltammetry

**Figure 4:**
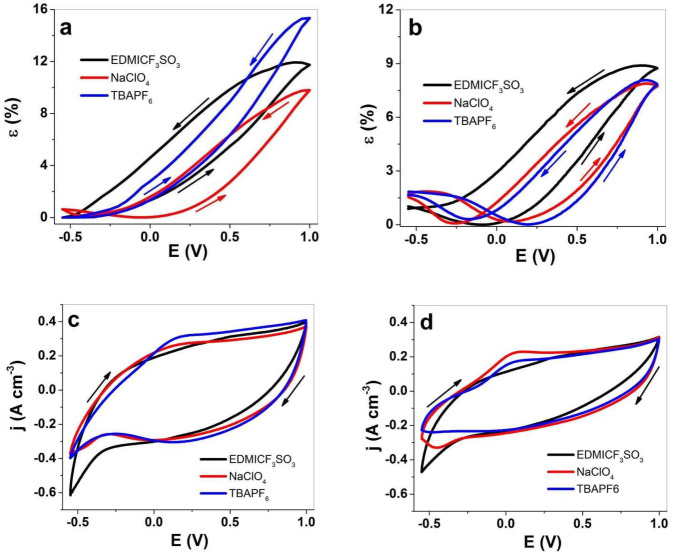
Cyclic voltammetry (scan rate 5 mV s^−1^) applied to PPy samples in EDMICF_3_SO_3_-PC (black curve), NaClO_4_-PC (red curve) and TBAPF_6_-PC (blue curve) electrolytes at potential range 1.0 V to −0.55 V showing strain ε of (**a**) PPy-PEO and (**b**) PPy/DBS against potential E. The current density potential curves for (**c**) PPy-PEO and (**d**) PPy/DBS samples. The arrows indicate the start and end points of the cycles.

The strain values at cyclic voltammetry against the applied potential of PPy-PEO of three electrolytes EDMICF_3_SO_3_-PC, NaClO_4_-PC and TBAPF_6_-PC are shown in Figure 4a, and those in comparison with PPy/DBS are shown in Figure 4b. The current density potential curves of PPy-PEO are presented in Figure 4c, and those of PPy/DBS are shown in Figure 4d. The charge density curves are shown in Figure S2a (PPy-PEO) and Figure S2b (PPy/DBS).

Linear strain of 15.4% was found for PPy-PEO (Figure 4a) in TBAPF_6_-PC, followed by 12% strain in EDMICF_3_SO_3_-PC and 10% for NaClO_4_-PC electrolytes with main expansion at oxidation. Only in the case of NaClO_4_-PC electrolyte, there was a small expansion of 0.7% strain at reduction, while all other electrolytes had only expansion at oxidation. The strain potential curves in Figure 4b for PPy/DBS revealed mixed-ion actuation for all applied electrolytes, with main expansion at oxidation of 8.8% for EDMICF_3_SO_3_-PC (1% strain at reduction), 7.9% for TBAPF_6_-PC and similar expansion at reduction in the range of 1.8% for NaClO_4_-PC electrolyte. The application of organic solvent in previous research [8] showed that the immobile DBS^−^ anions in PPy/DBS systems cannot dissociate the DBS^−^cation^+^ inside the PPy network in PC solvent, and during oxidation new places are occupied with solvated anions, causing the former cation-driven actuator reactions to change to anion-driven reactions. The strain at reduction shown in Figure 4b for most applied electrolytes is the reason for incorporating anions at oxidation which do not leave in full extent during reduction, leading to expansion at reduction with cation insertion [7]. In the case of PPy-PEO films, we assume the better ion conductivity (Figure 2b) led to faster egress of anions and solvent molecules during reduction with only small expansion found for NaClO_4_-PC electrolyte (Figure 3c, EDX spectroscopy, small amounts of Cl can be found at reduced state). Mixed actuation in conducting polymer actuators occurred in several studies [15,16], and the goal in general is to have only one ion that controls the actuation direction.

The current density potential curves for PPy-PEO (Figure 4c) reveal nearly 1.4 times higher current density in comparison to PPy/DBS (Figure 4d). The EDMICF_3_SO_3_-PC electrolyte for both PPy systems did not reveal any oxidation or reduction peaks, while for NaClO_4_-PC and TBAPF_6_-PC electrolytes an oxidation peak was found at 0.15 V for PPy-PEO (Figure 4c) and for PPy/DBS samples (Figure 4d) an oxidation peak at 0.05 V was found for same electrolytes. The charge density potential curves for PPy-PEO (Figure S2a) and PPy/DBS (Figure S2b) revealed for applied electrolytes closed loops which showed that the system is in “steady state”, meaning charging/discharging is in control [46] and no over-oxidation or over-reduction is taking place. The charge densities for PPy-PEO found an average of all applied electrolytes in the range of 73−80 C cm^−3^, nearly 1.4 times higher than those for PPy/DBS in the range of 52–57 C cm^−3^. To obtain more information on how the charge density contributes to the linear actuation properties, square potential steps of both PPy samples were conducted.

#### 3.2.2. Square Potential Steps

**Figure 5:**
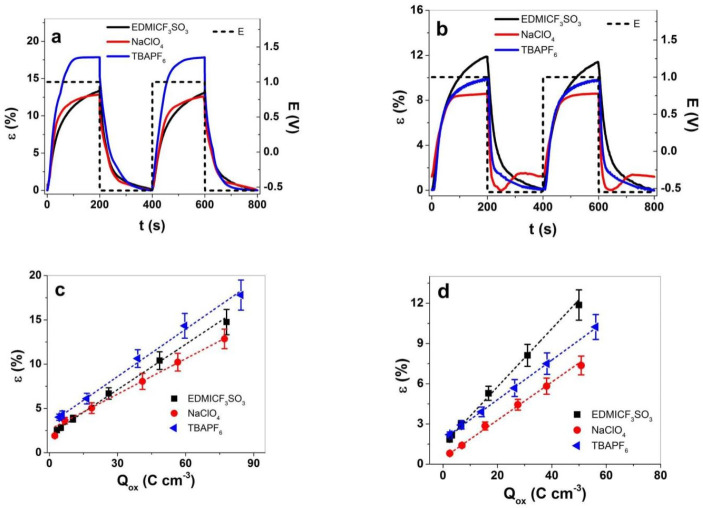
Square potential steps at applied frequency 0.0025 Hz (two subsequent cycles 3rd−4th) in potential range 1.0 V to −0.55 V (E, dashed line) in electrolytes EDMICF_3_SO_3_-PC (black curve), NaClO_4_-PC (red curve) and TBAPF_6_-PC (blue curve) showing strain of (**a**) PPy-PEO and (**b**) PPy/DBS films. The strain against charge densities in electrolytes EDMICF_3_SO_3_ (■), NaClO_4_ (●) and TBAPF_6_ (◄) for (**c**) PPy-PEO and (**d**) PPy/DBS films. The dashed lines represent the linear fit shown here for orientation only.

Square potential steps were applied on PPy samples at frequencies 0.0025 Hz to 0.1 Hz to determine the strain response in regard to frequencies (Figure S3a,b). At frequency 0.0025 Hz, two subsequent cycles of the three different electrolytes of PPy-PEO applied are shown in Figure 5a, and those for PPy/DBS are in Figure 5b. Conducting polymers are faradaic actuators [47], and the charge densities determine the linear actuation; hence, at higher charge densities more ions with solvent molecules enter and lead to higher reversible volume change at redox reaction. The results of strain vs. charge densities for PPy-PEO are presented in Figure 5c, and those for PPy/DBS are presented in Figure 5d.

Strain with main expansion at oxidation is shown in Figure 5a in the range of 18% for PPy-PEO in TBAPF_6_-PC electrolyte and 13% for EDMICF_3_SO_3_-PC and NaClO_4_-PC electrolytes. In the case of PPy/DBS (Figure 5b), the best strain at oxidation was found for EDMICF_3_SO_3_-PC in range of 12%, followed by 10% for TBAPF_6_-PC and 8.5% for NaClO_4_-PC electrolyte with expansion at reduction in range of 1.2% (overall strain reduced to 7.3%). The 1.8 times higher strain of PPy-PEO compared to PPy/DBS in TBAPF_6_-PC electrolyte is related to improved ion conductivity (nearly 1.3 times, Figure 2b), 1.8 times higher electronic conductivity (Table 1) where both factors lead to higher charge densities. Figure 5c,d presents the strain values against charge densities of PPy-PEO and PPy/DBS obtained from current density time curves over integration at each applied frequency. The charge densities of PPy-PEO in comparison to PPy/DBS were found in the range of 1.5 times higher for each applied electrolyte. Low frequency 0.0025 Hz (Figure S3a,b) was related to high charge density, revealing for both PPy films a nearly linear dependence of charge density on the strain of the faradaic actuators, shown as well from past research [48,49]. The solvation and size of the anions in the electrolyte need to be considered, whereas the fluorinated anions such as CF_3_SO_3_^−^ (EDMICF_3_SO_3_) and PF_6_^−^ (TBAPF_6_) are considered very weakly solvated [50] and expected to move as a single unity. We assume that having anions that move without a solvation shell led to faster ion ingress/egress and therefore better reversible volume change. The anion ClO_4_^−^ is solvated with 1.7–2.4 PC molecules [51], leading besides osmotic pressure [6] to additional solvent transport in PPy films at oxidation, which has an effect on ion mobility.

**Figure 6:**
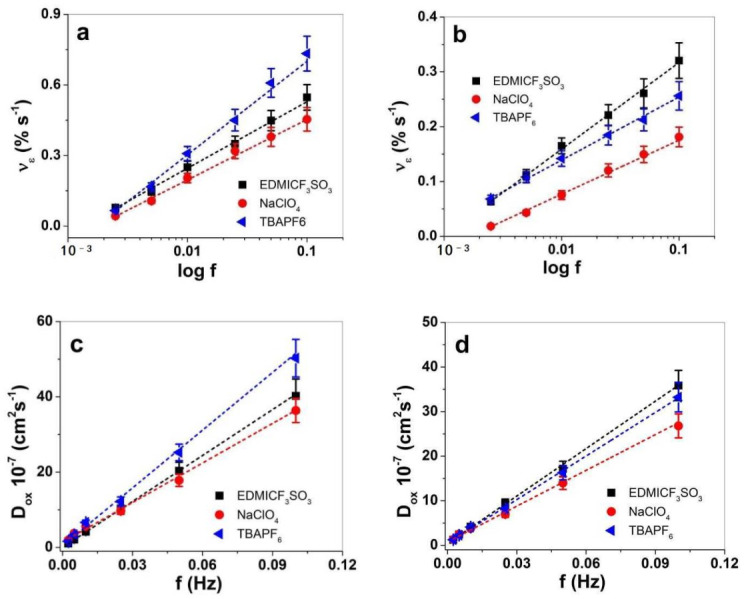
Square potential steps of PPy at applied potential range 1.0 V to −0.55 V and frequencies 0.0025 Hz to 0.1 Hz in propylene carbonate solvent with salts EDMICF_3_SO_3_ (■), NaClO_4_ (●) and TBAPF_6_ (◄) showing the strain rate ν_e_ against logarithmic scale of frequencies of (**a**) PPy-PEO and (**b**) PPy/DBS. The diffusion coefficients at oxidation D_ox_ determined from Equations (1) and (2) of PPy-PEO are shown in (**c**) and those of PPy/DBS presented in (**d**) against frequencies f. The dashed lines represent the linear fit and are shown for orientation only.

The actuation rate ν against applied frequencies is presented in Figure 6a,b, and the diffusion coefficients at oxidation are shown in Figure 6c,d.

The strain rate of PPy-PEO (Figure 6a) revealed for all applied electrolytes nearly double values in comparison to PPy/DBS (Figure 6b). The best strain rates of PPy-PEO were found for TBAPF_6_-PC electrolyte, while for PPy/DBS films the electrolyte EDMICF_3_SO_3_-PC dominated. For future application of PPy-based linear actuators in soft robotics or smart textiles, the strain rate will give the limitation where such composites suit the best. The diffusion coefficient at oxidation is another speed value of ion diffusion having the same tendency of PPy-PEO (Figure 6c) and PPy/DBS films (Figure 6d) that with increasing frequency the diffusion coefficient increases as well. The main reason for such phenomena relies on the different process taking place in PPy films, where at low frequencies there is more time at oxidation leading to higher reaction charges that induce reaction-driven conformational and structural changes [52] such as relaxation/swelling process [53] following the ESCR model. At shorter time (higher frequencies), only swelling took place, leading to higher diffusion coefficients. In the case of PPy-PEO linear actuators, the diffusion coefficient at oxidation (Figure 6c) was found best in the electrolyte TBAPF_6_-PC, followed by EDMICF_3_SO_3_-PC, and lowest values were shown for NaClO_4_-PC. In the case of PPy/DBS (Figure 6d) the diffusion coefficients at oxidation were found enhanced for the electrolyte EDMICF_3_SO_3_-PC, slightly reduced for TBAPF_6_-PC and lowest for NaClO_4_-PC. Table 2 shows the comparison of PPy-PEO and PPy/DBS in terms of strain (Figure S3a,b), the strain rate (Figure 6a,d) and the diffusion coefficients at oxidation at applied frequency 0.1 Hz in different electrolytes.

**Figure 7:**
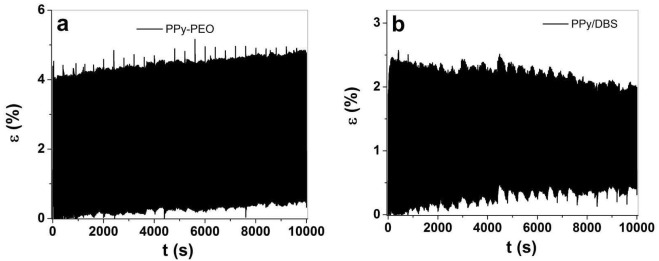

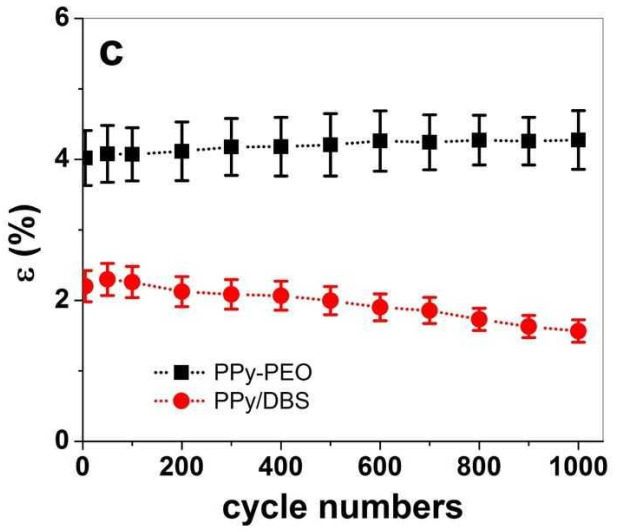
Square potential steps at 0.1 Hz in TBAPF_6_-PC electrolyte at potential range 1.0 V to −0.55 V showing strain ε against time t of (**a**) PPy-PEO and (**b**) PPy/DBS. The strain against cycle numbers 5–1000 of PPy-PEO (∙∙■∙∙) and PPy/DBS (∙∙●∙∙) are presented in (**c**).

The strain at 0.1 Hz in TBAPF_6_-PC (Table 2) of PPy-PEO linear actuators revealed nearly 1.8 times higher values in strain rates and 1.5 times higher diffusion coefficients in comparison to PPy/DBS. The second best actuation properties are found for EDMICF_3_SO_3_-PC electrolyte with nearly 1.4 times higher strain, 1.7 times higher strain rates and 1.1 times higher diffusion coefficients for PPy-PEO films. The electrolyte NaClO_4_-PC electrolyte (Table 2) revealed better values in PPy-PEO due to mainly anion-driven reaction, while mixed actuation was detected in PPy/DBS (Figure 5b), which lowered the diffusion coefficient 1.3 times. So far, in view of the best electrolyte with the best actuation values, the electrolyte TBAPF_6_-PC stood out for PPy-PEO films. In view of reliable actuators regarding which electrolyte has the best response, long-term measurements were performed at 0.1 Hz frequency up to 1000 cycles. The results are shown in Figure 7.

The long-term measurements revealed that small creep appeared in both PPy films, in the range of 0.28% for PPy-PEO (Figure 7a) and 0.34% (Figure 7b) for PPy/DBS. The creep is not fully understood, but it is basically a shifting of the starting position partly explained by irreversible charging [54]. Other research was made in the past to draw high creep development to high loads during reversible redox cycles of PPy linear actuators [55]. Figure 7c shows the strain against cycle numbers with increasing strain for PPy-PEO and decreasing strain for PPy/DBS films. The linear strain profiles shown in Figure S4a for PPy-PEO show a slight increase in strain (4.02% at cycle 5 and 4.27% at cycle 1000), while for PPy/DBS the strain decreased and was found as 2.2% at cycle 5 and 1.56% at cycle 1000 (Figure S4b). PPy-based composites are faradaic actuators; therefore, the charge density increases if strain increases, as shown in Figure S4c for PPy-PEO films. In the case of PPy/DBS, the strain decreased in long-term measurements, which is also reflected in the lower charge density shown in Figure S4c. The general conclusion is that the electrolyte TBAPF_6_-PC, being preferred in PPy linear actuators [7], is also shown as beneficial with the best strain, strain rate and long-term cycling in PPy-PEO composite films, suitable for future consideration in artificial muscle-like applications.

## 4. Conclusions

Avoiding mixed-ion actuation even only to a small extent is needed to assure controllable linear actuation in one direction (here oxidation). PPy-PEO (5 wt.% PEO) and PPy/DBS films’ linear actuation properties were compared by applying three electrolytes such as EDMICF_3_SO_3_, NaClO_4_ and TBAPF_6_ in PC solvent. In the case of PPy/DBS films, all applied electrolytes revealed mixed-ion actuation in cyclic voltammetry. PPy-PEO linear actuators revealed expansion at oxidation for all applied electrolytes, whereas the electrolyte TBAPF_6_-PC stood out from all, having 1.8 times higher electronic conductivity, best strain of 18%, 2 times higher strain rate and 1.5 times higher diffusion coefficients. One of the reasons for the better performance of PPy-PEO films is shown in the better doping state of the films directly after polymerization (Raman spectroscopy) and the 1.3 times higher ion conductivity (EIS measurements) in PC solvent in comparison to PPy/DBS. The selection of best performance in chosen electrolyte revealed that PPy-PEO composites showed a nearly stable strain in the range of 4% in long-term cycling of 1000 cycles (0.1 Hz, TBAPF_6_). The controllable linear actuation of PPy-PEO is envisaged in applications of soft robotics and smart textiles where large strain and longevity are needed.

## Figures and Tables

**Table 1:** Electronic surface conductivities σ_e_ of PPy/DBS and PPy-PEO films directly after polymerization (pristine) and after actuation studies in different electrolytes.

PPy Films	Pristine (S cm^−1^)	EDMICF_3_SO_3_-PC (S cm^−1^)	NaClO_4_-PC (S cm^−1^)	TBAPF_6_-PC (S cm^−1^)
PPy/DBS.	2.1 ± 0.2	6.4 ± 0.7	4.6 ± 0.4	6.2 ± 0.6
PPy-PEO	3.9 ± 0.8	11.4 ± 0.9	6.3 ± 0.5	13.1 ± 1.2

**Table 2:** Strain ε, strain rate ν_ε_ and diffusion coefficients at oxidation D_ox_ of PPy-PEO and PPy/DBS in electrolytes EDMICF_3_SO_3_-PC, NaClO_4_-PC and TBAPF_6_-PC at frequency 0.1 Hz.

Electrolytes in PC	ε (%)		ν_ε_ (% s^−1^)		D_ox_ 10^−7^ (cm^2^ s^−1^)	
	PPy-PEO	PPy/DBS	PPy-PEO	PPy/DBS	PPy-PEO	PPy/DBS
EDMI-CF_3_SO_3_	2.54 ± 0.22	1.85 ± 0.15	0.55 ± 0.05	0.32 ± 0.03	40 ± 4.5	35 ± 3.4
NaClO_4_	1.91 ± 0.20	0.8 ± 0.07	0.45 ± 0.04	0.18 ± 0.02	36 ± 3.5	27 ± 2.4
TBAPF_6_	4.0 ± 0.35	2.2 ± 0.2	0.73 ± 0.07	0.26 ± 0.03	50 ± 4.8	33 ± 3.2

## Data Availability

The data presented in this study are available on request from the Corresponding author.

## Supplementary Materials

The following supporting information can be downloaded at: https://www.mdpi.com/article/10.3390/ma15020540/s1, Sheme S1: Images of measurement set-up showing the LAS (linear actuation staging) connected with the forces sensor, potentiostat, Voltmeter and LAS controller. The measurement cell with electrolyte consist of a three electrode cell included the PPy film as working electrode (WE) clamped between force sensor and fixed arm (with gold contacts), reference electrode (RE, Ag/AgCl (3MKCl)) and counter electrode (CE, platinum sheet). The potentiostat with the LAS is controlled over a home-made software translate the electrochemical signals and change of volume (strain or stress) of the PPy films in real time; Figure S1: EDX spectroscopy of PPy/DBS linear films at oxidation (black line, 1.0V, 1min) and at reduction (red line, -0.55V, 1 min) obtained from cross-section image after actuation cycles in different electrolytes presenting in a: EDMICF_3_SO_3_-PC, b: NaClO_4_-PC and c: TBAPF_6_-PC; Figure S2: Charge density potential curve at cyclic voltammetry (scan rate 5 mV s^−1^, 3^rd^ cycle) of a: PPy-PEO/DBS and b: PPy/DBS films in EDMICF_3_SO_3_-PC (black curve), NaClO_4_-PC (red curve) and TBAPF_6_-PC (blue curve) electrolytes at potential range 1.0V to -0.55V. The arrows indicate start and end point of the cycle; Figure S3: Square potential steps of PPy samples in EDMICF_3_SO_3_-PC (■,) NaClO_4_-PC (●) and TBAPF_6_-PC (◄) electrolyte at potential range 1.0V to -0.55V showing in a: PPy-PEO/DBS and b: PPy/DBS linear films; Figure S4: Square potential steps at 0.1Hz in TBAPF_6_-PC electrolyte showing cycle 5 (black line), cycle 500 (red line) and cycle 1000 (blue line) at applied potential range E (dashed line, 1.0V to -0.55V) showing strain against time in a: of PPy-PEO/DBS and b: PPy/DBS linear films. The charge density at oxidation Q_ox_ against cycle numbers of PPy-PEO/DBS (∙∙■∙∙) and PPy/DBS (∙∙●∙∙) is presented in (c).
