# Supplementary material for: Polypyrrole Polyethylene Composite for Controllable Linear Actuators in Different Organic Electrolytes
Source: Materials (Basel). 2022 Jan 12;15(2):540. doi: 10.3390/ma15020540 (PMC8781785; doi:10.3390/ma15020540)
Supplement: Supplementary file 1 [file materials-15-00540-s001.zip › materials-1477950-supplementary.pdf]

materials

Supplementary Materials

Polypyrrole Polyethylene Composite for Controllable Linear Actuators in Different Organic Electrolytes

Nguyen Quang Khuyen [1], Ngoc Tuan Nguyen [2] and Rudolf Kiefer [1,*]

[1] Conducting polymers in composites and applications Research Group, Faculty of Applied Sciences, Ton Duc Thang University, Ho Chi Minh City 700000, Vietnam; nguyenquangkhuyen@tdtu.edu.vn
[2] Faculty of Applied Sciences, Ton Duc Thang University, Ho Chi Minh City 700000, Vietnam; nguyenngoctuan@tdtu.edu.vn
* Correspondence: rudolf.kiefer@tdtu.edu.vn; Tel: 886-905-60-55-15

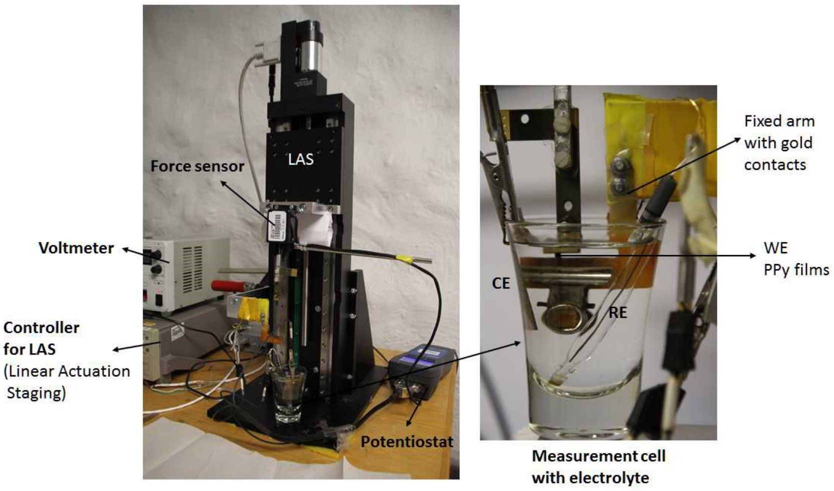

.

Scheme S1. Images of measurement set-up showing the LAS (linear actuation staging) connected with the forces sensor, potentiostat, Voltmeter and LAS controller. The measurement cell with electrolyte consist of a three electrode cell included the PPy film as working electrode (WE) clamped between force sensor and fixed arm (with gold contacts), reference electrode (RE, Ag/AgCl (3MKCl)) and counter electrode (CE, platinum sheet). The potentiostat with the LAS is controlled over a homemade software translate the electrochemical signals and change of volume (strain or stress) of the PPy films in real time.

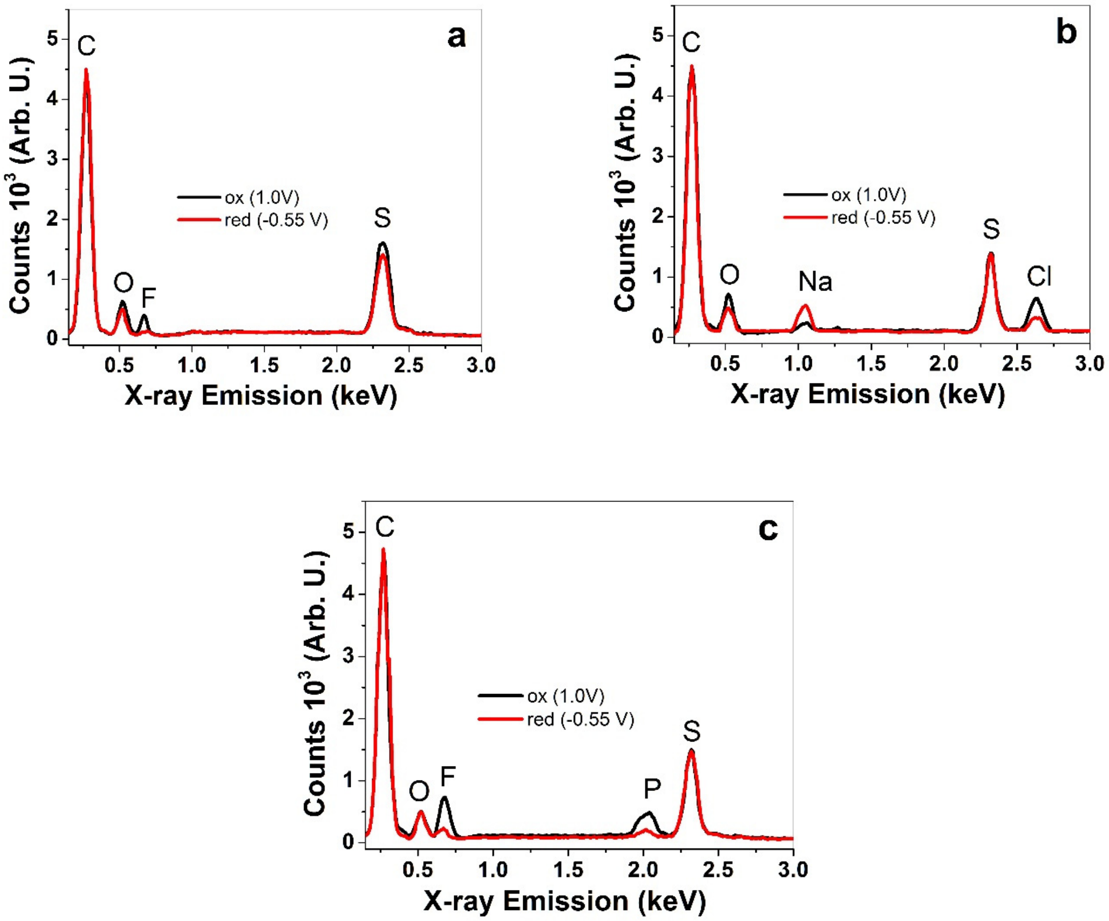

Figure S1. EDX spectroscopy of PPy/DBS linear films at oxidation (black line, 1.0V, 1min) and at reduction (red line, -0.55V, 1 min) obtained from cross-section image after actuation cycles in different electrolytes presenting in a: EDMICF$_3$SO$_3$-PC, b: NaClO$_4$-PC and c: TBAPF$_6$-PC.

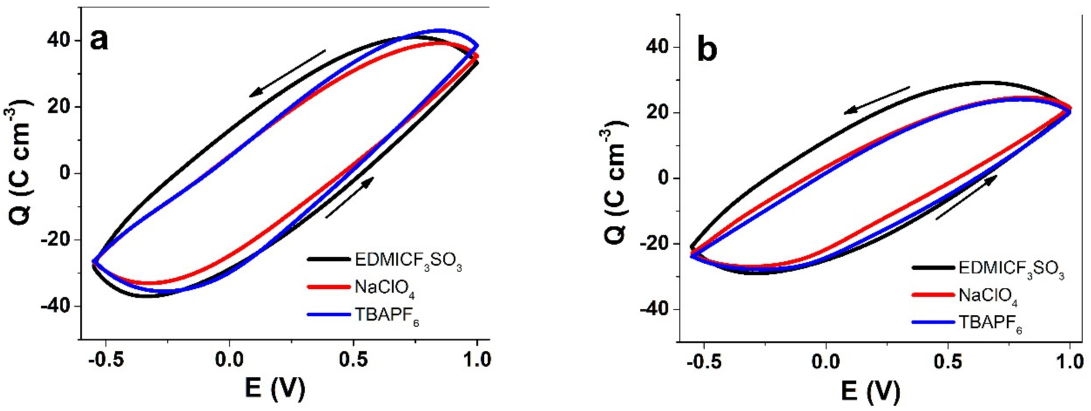

Figure S2. Charge density potential curve at cyclic voltammetry (scan rate 5 mV s^{-1}, 3rd cycle) of a: PPy-PEO/DBS and b: PPy/DBS films in EDMICF$_3$SO$_3$-PC (black curve), NaClO$_4$-PC (red curve) and TBAPF$_6$-PC (blue curve) electrolytes at potential range 1.0V to -0.55V. The arrows indicate start and end point of the cycle.

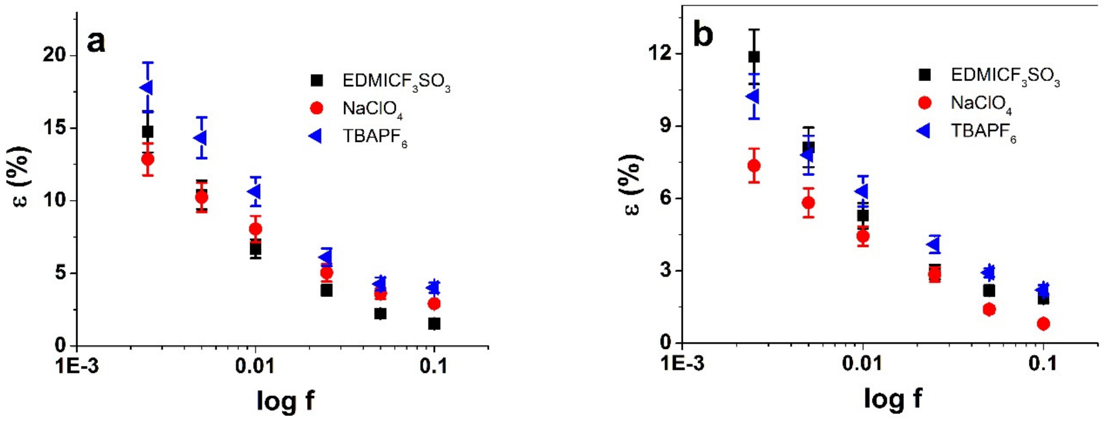

Figure S3. Square potential steps of PPy samples in $EDMICF_3SO_3$-PC (■,) $NaClO_4$-PC (●) and $TBAPF_6$-PC (◀) electrolyte at potential range 1.0V to -0.55V showing in a: PPy-PEO/DBS and b: PPy/DBS linear films.

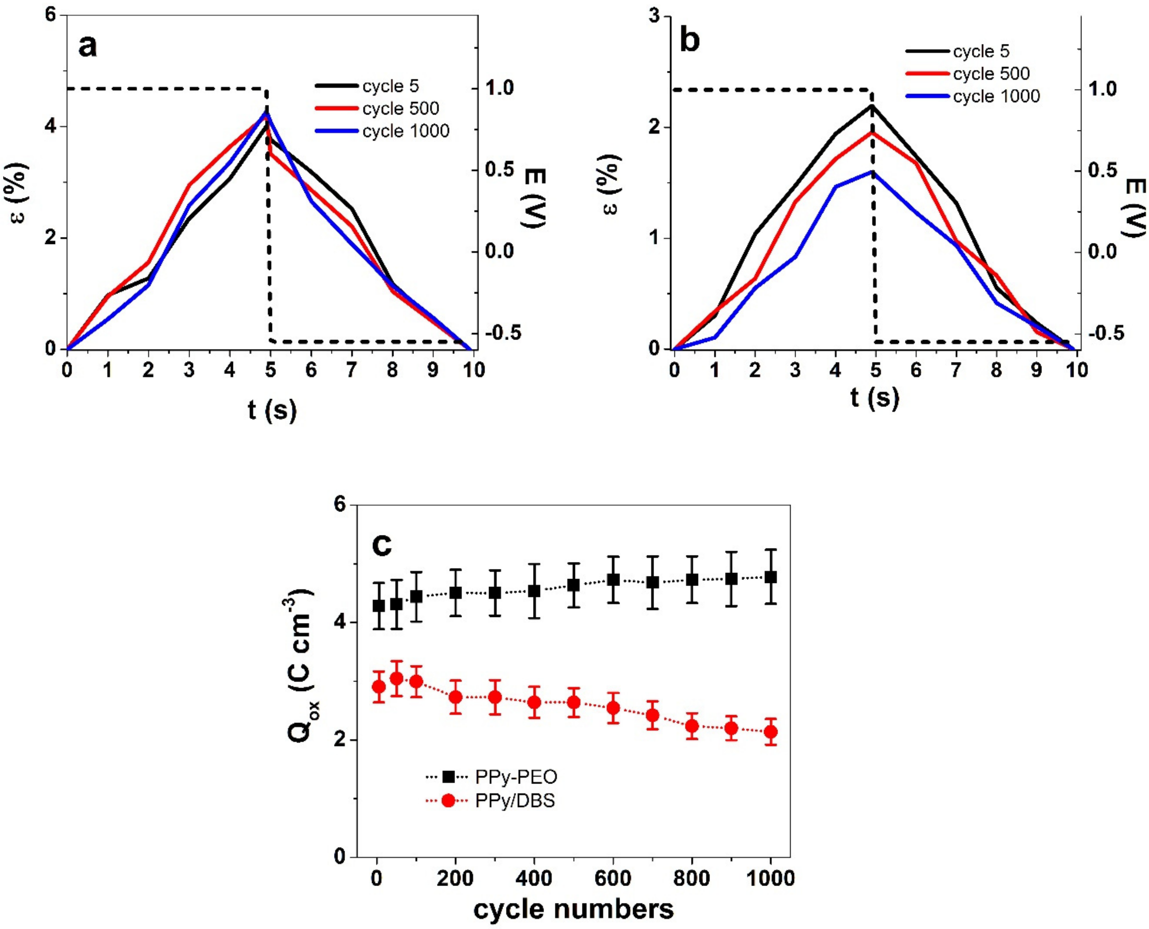

Figure S4. Square potential steps at 0.1Hz in $TBAPF_6$-PC electrolyte showing cycle 5 (black line), cycle 500 (red line) and cycle 1000 (blue line) at applied potential range E (dashed line, 1.0V to -0.55V) showing strain against time in a: of PPy-PEO/DBS and b: PPy/DBS linear films. The charge density at oxidation Q_{ox} against cycle numbers of PPy-PEO/DBS (··■··) and PPy/DBS (··●··) is presented in (c).
